# [^68^ Ga]Ga-FAPI uptake correlates with the state of chronic kidney disease

**DOI:** 10.1007/s00259-021-05660-1

**Published:** 2022-01-06

**Authors:** Patrick Conen, Francesca Pennetta, Katharina Dendl, Fabian Hertel, Andreas Vogg, Uwe Haberkorn, Frederik L. Giesel, Felix M. Mottaghy

**Affiliations:** 1 Department of Nuclear Medicine, University Hospital RWTH Aachen University, Pauwelsstr. 30, 52074 Aachen, Germany; 2 Center of Integrated Oncology (CIO), Universities of Aachen, Bonn, Cologne, and Duesseldorf, Cologne, Germany; 3 Department of Internal Medicine, Klinikum Garmisch-Partenkirchen, Auenstraße 6, 82467 Garmisch-Partenkirchen, Germany; 4 Clinical Cooperation Unit Nuclear Medicine, German Cancer Research Center (DKFZ), Im Neuenheimer Feld 280, 69120 Heidelberg, Germany; 5 Department of Nuclear Medicine, University Hospital Heidelberg, Im Neuenheimer Feld 400, 69120 Heidelberg, Germany; 6 Translational Lung Research Center Heidelberg (TLRC), German Center for Lung Research (DZL), Heidelberg, Germany; 7 Department of Nuclear Medicine, Medical Faculty Heinrich-Heine-University, University Hospital Düsseldorf, Moorenstraße 5, 40225 Düsseldorf, Germany; 8 Department of Radiology and Nuclear Medicine, Maastricht University Medical Center (MUMC+), P. Debeylaan 25, 6229 HX Maastricht, P.O. Box 5800, 6202 AZ Maastricht, The Netherlands

**Keywords:** Fibroblast-activation-protein, Kidney fibrosis, FAPI PET/CT, DOTATOC PET/CT, PSMA PET/CT

## Abstract

**Purpose:**

Kidney fibrosis leads to a progressive reduction in kidney function ultimately resulting in kidney failure. Diagnostic tools to detect kidney fibrosis are all invasive in nature requiring kidney biopsies with subsequent histological validation. In this retrospective study, the diagnostic value of three different radiotracers for the noninvasive prediction of kidney fibrosis was analyzed, taking into account the glomerular filtration rate (GFR) and the intra-renal parenchymal radiotracer uptake.

**Methods:**

In 81 patients receiving either one of the following molecular imaging probes, [^68^ Ga]Ga-FAPI, [^68^ Ga]Ga-PSMA, or [^68^ Ga]Ga-DOTATOC, kidney function parameters were correlated with SUVmax and SUVmean of the renal parenchyma and background activity measured in lung parenchyma, myocardium, gluteal muscle, and the abdominal aorta. Patients were clustered according to their grade of chronic kidney disease (CKD), and a regression analysis and one-way ANOVA were conducted in this retrospective analysis.

**Results:**

We found a negative correlation between GFR and [^68^ Ga]Ga-FAPI uptake for both SUVmax and SUVmean values, whereas background activity showed no correlation with GFR. [^68^ Ga]Ga-DOTATOC and [^68^ Ga]Ga-PSMA did not correlate between CKD stage and intra-renal parenchymal radiotracer uptake. Only [^68^ Ga]Ga-PSMA background activity exhibited a positive correlation with GFR suggesting an unspecific binding/retention potentially due to longer circulation times.

**Conclusion:**

There is a significant negative correlation between renal parenchymal [^68^ Ga]Ga-FAPI uptake and GFR, which was not the case for [^68^ Ga]Ga-DOTATOC and [^68^ Ga]Ga-PSMA. This correlation suggests a specific binding of FAPI rather than a potential unspecific retention in the renal parenchyma, underlining the potential value of [^68^ Ga]Ga-FAPI for the noninvasive quantitative evaluation of kidney fibrosis.

## Introduction

Kidney fibrosis is a chronic disease with the risk of a fast deterioration towards kidney failure and need for dialysis and a higher cardiovascular risk. Chronic kidney disease (CKD) is defined as kidney damage or impaired glomerular filtration rate ((GFR) < 60 mL/min/1.73 m^2^) for a duration of at least 3 months, irrespective of cause [1]. Therefore, not only the GFR but also albuminuria and diagnostic tools like ultrasound and computed tomography to evaluate the kidney structure are important components in diagnosing CKD [2].

An early diagnosis is vital in order to achieve a better prognosis and an early therapeutic intervention/treatment of the causing disease (e.g., hypertension, diabetes mellitus, chronic glomerulonephritis).

The current reference diagnostic tools for the detection of renal fibrosis are invasive and require the execution of a biopsy followed by histological confirmation. The latter has its associated risks, is highly demanding for the patient, and time-consuming. Therefore, the need grows for a less invasive and faster diagnostic modality.

Previous attempts to noninvasively detect kidney fibrosis have been done with functional MRI which has not been amplified in clinical use yet. Possible MRI target structures or processes in fibrotic kidneys are elastin imaging [3], the collagen-binding adhesion protein CNA35 [4], deoxyhemoglobin levels, diffusion-weighted imaging to quantify deposition of extracellular collagen, and microvascular perfusion [5].

The feasibility of PET imaging in kidney fibrosis detection has been evaluated. The predominantly applied PET tracer [^18^F]FDG is able to identify kidney cyst infections and showed also a potential in diagnosing acute rejection after kidney transplantation [6]. The potential of different imaging approaches for kidney diseases has recently been reviewed [7].

A recent study by Zhou et al. has compared the degree of renal fibrosis determined by kidney biopsy with the tracer uptake of [^68^ Ga]Ga-FAPI in thirteen patients. An increased tracer accumulation has been found to be correlated with a higher degree of kidney fibrosis as defined by immunohistopathology [8].

We set up a slightly different “real-world” study evaluating the potential value of FAPI and other molecular imaging probes for the detection and determination of different degrees of CKD. Next to [^68^ Ga]Ga-FAPI-04 (fibroblast activation protein inhibitor), also [^68^ Ga]Ga-PSMA (prostate specific membrane antigen) and [^68^ Ga]Ga-DOTATOC (DOTA-Phe1-Tyr3-Octreotid) were investigated.

It is known that in the early stages of renal fibrosis, an accumulation of fibroblasts occurs in the renal parenchyma [9]. Several studies have shown that the radiotracer [^68^ Ga]Ga-FAPI binds to fibroblast-activation-protein (FAP) in fibroblasts of tumor tissue [10]. This binding of FAP also occurs in non-tumor tissue such as in hepatic fibrotic samples [11]. The binding principle has been applied in this study to analyze whether the expression of FAP binding of [^68^ Ga]Ga-FAPI in renal-parenchymal tissue correlates with the CKD stage of the patients. The abovementioned study (Zhou et al., 2021) has already been able to show that the concept of FAP expression works also for CKD; however, there was no comparison to a healthy cohort included.

The second radiotracer analyzed in this study is [^68^ Ga]Ga-DOTATOC. The compound is an octreotide DOTA-conjugated peptide which has the ability to bind to somatostatin receptors (SSTRs) with a strong tendency towards the SST-2 receptor [12]. It has been shown that diseased kidneys have an increased somatostatin receptor expression [13]. On this basis, we investigated whether a correlation exists between the binding of [^68^ Ga]Ga-DOTATOC in renal parenchyma and the stage of kidney disease, thus whether this radiotracer has a potential diagnostic strength for the detection of kidney fibrosis.

Prostate-specific membrane antigen (PSMA) normally binds to prostate tissue, showing an increased expression in tumor tissues [14]. Thus, it has been widely accepted as a target for imaging and radionuclide therapy of prostate cancer [15]. Additionally, PSMA physiologically binds to cells of the proximal tubules of the kidneys, as well as salivary glands and brain tissue [16]. Therefore, its physiological accumulation in the kidneys could potentially serve as a diagnostic correlate for kidney disease.

## Study and methods

### Study design

We retrospectively analyzed PET data of a total of 81 patients who presented to the nuclear medicine department of the RWTH Aachen University hospital or the nuclear medicine department of the Heidelberg University hospital between August 2017 and October 2020. Sixteen patients received [^68^ Ga]Ga-FAPI-04 and four patients [^68^ Ga]Ga-FAPI-46, of which 15 were male and 5 were female, with a mean age of 69.2 years (some of these patients have already been part of other investigations with a different scope [10, 24]). A total of 34 patients received the [^68^ Ga]Ga-PSMA radiotracer, all male patients with a mean age of 73 years. [^68^ Ga]Ga-DOTATOC was employed in 27 patients, 16 males and 11 females with a mean age of 64 years. Further information on the total number of patients and patient characteristics are displayed in Table 1, and for each radiotracer, the number of patients with the respective stage is listed.

**Table 1:** Total number of patients of each CKD category in the different radiotracer groups

GFR	[^68^ Ga]Ga-FAPI	[^68^ Ga]Ga-PSMA	[^68^ Ga]Ga-DOTATOC	Total
> *90*	8	*8*	*10*	*26*
*60–89*	*3*	*17*	*10*	*30*
*30–59*	*7*	*7*	*6*	*20*
< *30*	*2*	*2*	*1*	*5*
*Total*	*20*	*34*	*27*	*81*

All patients gave their informed consent for their clinical data to be used for retrospective analysis and the study got approval from both ethics committees at the RWTH Aachen University Hospital and Heidelberg University hospital. The necessary clinical data, such as laboratory values, patient history, and PET/CT imaging, were retrieved from the RWTH Aachen University hospital and Heidelberg University hospital database. The laboratory values were determined by the Laboratory Diagnostic Center (LDZ) of the RWTH Aachen University hospital and the Central Laboratory of the Heidelberg University hospital. Inclusion criteria included the use of the radiotracer in a recent PET/CT-scan (from August 2017 up to and including October 2020) in the department of nuclear medicine of the RWTH Aachen University hospital and Heidelberg University hospital. Exclusion criteria were the inability to provide recent imaging or laboratory values (GFR and creatinine values older than 3 months) for the determination of CKD stage. Furthermore, patients were excluded, if they underwent a nephrectomy prior to the image analysis or showed signs of obstruction of the ureteral system.

### PET/CT imaging and radiotracer synthesis

Synthesis and labeling of [^68^ Ga]Ga-FAPI-04 and [^68^ Ga]Ga-FAPI-46 have already been described previously [17, 18]. The PET/CT imaging in Heidelberg for [^68^ Ga]Ga-FAPI was performed analogously to the PET/CT protocol previously described by Kratochwil et al. [10] using a Biograph mCT Flow scanner (Siemens). Following the regulations of the German Pharmaceuticals Act §13(2b), the indication for the exam and labeling of the FAPI tracers was done under the direct responsibility of the applying physician.

The injected activity for the [^68^ Ga]Ga-FAPI examinations was calculated using a range of 113–340 MBq injected activity. The PET scans were started 1 h after injection, and the patients were examined in craniocaudal direction.

Patients received 2 MBq/kg body weight either [^68^ Ga]Ga-DOTATOC or [^68^ Ga]Ga-PSMA (University Hospital RWTH Aachen) and were examined 60 min after injection. PET/CT acquisition was performed on a Philips Gemini TF 16 PET/CT (Philips Medical Systems, Best, The Netherlands). Patients were examined in craniocaudal orientation for [^68^ Ga]Ga-DOTATOC and caudocranial orientation for [^68^ Ga]Ga-PSMA with their arms raised to decrease beam-hardening artefacts. First a low-dose whole body CT from the base of the skull to the upper thigh was performed without contrast medium for attenuation correction purposes. CT parameters for the low-dose unenhanced CT were used analogue to the protocol by Behrendt et al. [19].

The ^68^ Ga-PSMA-HBED-CC tracer for the PET/CT scans was produced by the in-house radiopharmacy using a previously reported method [20]. As [^68^ Ga]Ga-DOTATOC, it was synthesized using the cassette-based synthesis module GRP-3 V from Scintomics [21].

### Clinical PET/CT imaging and data analysis

The evaluation of all the images for each radiotracer was carried out individually by a single observer with numerous years of experience in the field of nuclear medicine. This was done in order to ensure no inter-observer variation which could decrease the accuracy in the evaluation of the images. Moreover, the image analysis was carried out before the determination of the patient’s CKD stage and GFR by the observer. This was done to ensure the absence of any bias in the evaluation of the images.

**Fig. 1:**
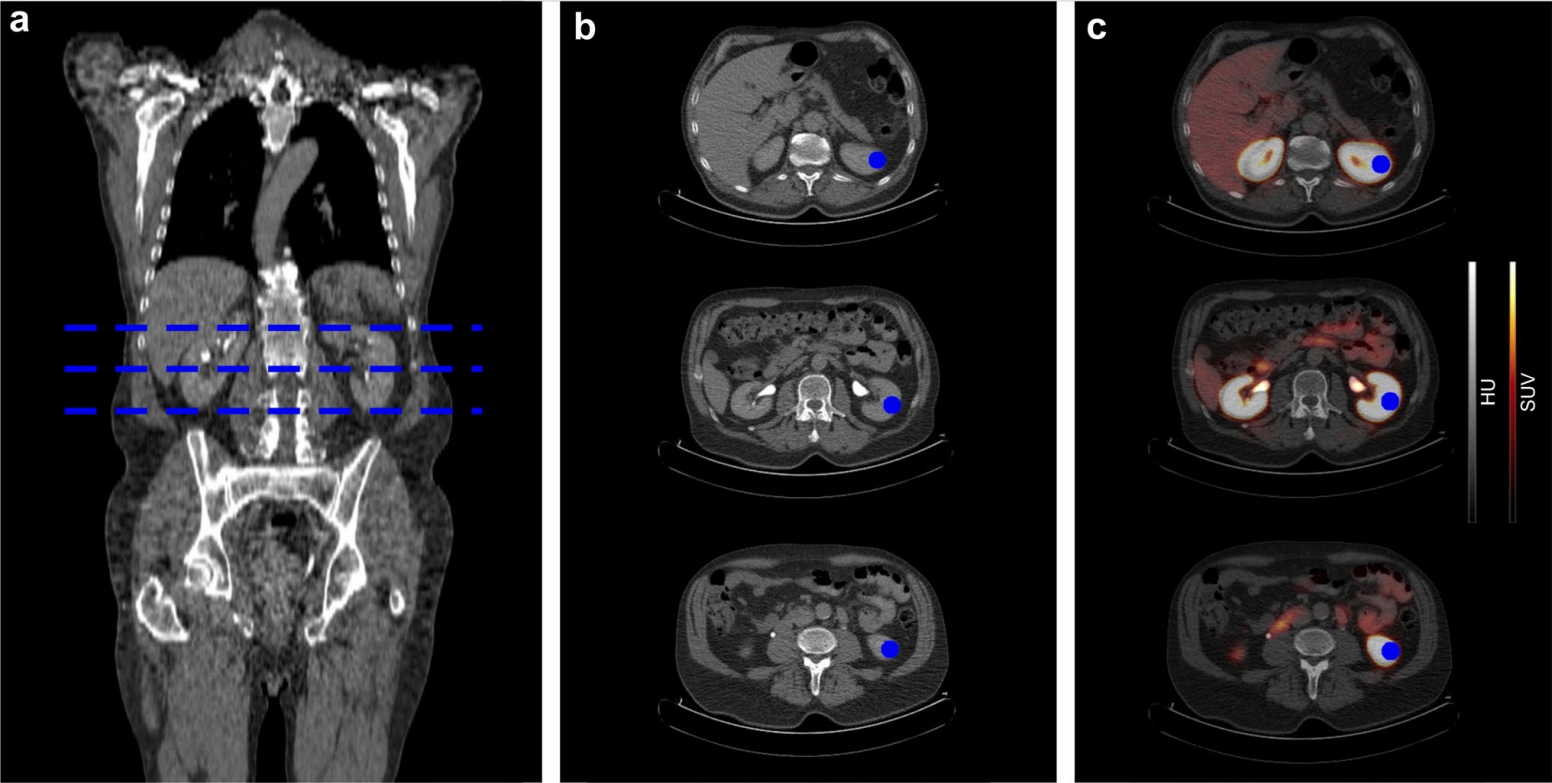
Example of the measurement of renal tracer uptake in a patient who received a PSMA-PET/CT. Representative illustration of the CT-based ROI placement in three planes of the kidney (blue areas). For all three radiotracers, ROI drawing was performed accordingly. Renal uptake was quantified using SUVmax and SUVmean at the three depicted locations. **a** Whole-body CT scan, blue intermittent lines showing the position of 3 axial sections. **b** CT sections with drawn ROIs (blue). **c** PET/CT hybrid section with superimposed ROIs (blue)

The focus of this study was to analyze the PET images, in order to visualize the uptake of the radiotracers in the pre-determined locations. For the purpose of a better anatomical visualization, the PET images were fused with a low-body-dose CT. Renal uptake of all three radiotracers was quantified using SUVmax (standardized uptake value maximum) and SUVmean (standardized uptake value mean) at separate locations of the renal parenchyma (superior, middle, and inferior renal cortex) as illustrated in Fig. 1. Background activity was measured in lung parenchyma, myocardium, and gluteal muscle and includes blood pool activity which was measured in the abdominal aorta at the level of the renal arteries.

### Statistical analysis

Statistical analyses were performed using SPSS 22.0 (SPSS Inc., Chicago, IL). Normality test was conducted to the quantitative variable. All groups showed a normal distribution. The association between SUVmax respectively SUVmean and GFR was analyzed by linear regression analysis and one-way analysis of variance (ANOVA).

We used a Student’s *t* test to compare the age structure of the different patient groups.

## Results

### *[*^*68*^* Ga]Ga-FAPI-PET/CT*

**Fig. 2:**
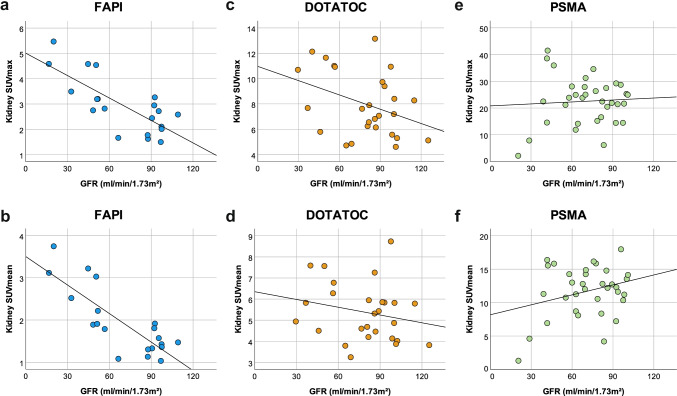
Comparison of linear regression analyses of the correlation between kidney SUV and GFR for three radiotracers. [^68^ Ga]Ga-FAPI (**a**, b), [^68^ Ga]Ga-DOTATOC (**c**, **d**), and [^68^ Ga]Ga-PSMA (**e**, **f**); top row kidney SUVmax and bottom row kidney SUVmean scans correlated with the GFR. Dots represent measurements of individual patients and the calculated regression line is depicted. The abscissa indicates the GFR, and the ordinate depicts the SUVmax (top row) or SUVmean (bottom row), respectively. The R^2^ values (coefficient of determination of the regression) are 0.572 (**a**), 0.664 (**b**), 0.134 (**c**), 0.049 (**d**), 0.004 (**e**), and 0.082 (**f**)

Figure 2a and b show the relationship between the mean SUVmax and SUVmean values (upper pole, middle, lower pole) and the GFR of patients who received a [^68^ Ga]Ga-FAPI PET/CT scan. Both regression lines indicate a clear inverse correlation between the GFR and the tracer uptake with a higher tracer uptake correlating with a poorer GFR (R^2^ value of 0.572 and 0.664, respectively). The highest individual values were measured in a patient in CKD stage IV. The correlation found in the regressions lines could also be confirmed using one-way ANOVA (Table 2), which showed a significant relationship between tracer accumulation and GFR.

**Table 2:** [^68^ Ga]Ga-FAPI/[^68^ Ga]Ga-DOTATOC/[^68^ Ga]Ga-PSMA ANOVA results for SUVmax and SUVmean evaluation; *s* significant, *ns* nonsignificant

	df	*F*	*p*	η^2^	Significance
[^68^ Ga]Ga-FAPI					
SUVmax kidney	1, 18	24.101	< 0.001	0.572	s
SUVmax background	1, 18	3.687	0.071	0.170	ns
SUVmean kidney	1, 18	35.553	< 0.001	0.664	s
SUVmean background	1, 18	1.567	0.227	0.080	ns
[^68^ Ga]Ga-DOTATOC					
SUVmax kidney	1, 25	3.880	0.060	0.134	ns
SUVmax background	1, 25	< 0.001	0.998	< 0.001	ns
SUVmean kidney	1, 25	1.294	0.266	0.049	ns
SUVmean background	1, 25	0.236	0.631	0.009	ns
[^68^ Ga]Ga-PSMA					
SUVmax kidney	1, 32	0.117	0.734	0.004	ns
SUVmax background	1, 32	10.700	0.003	0.251	s
SUVmean kidney	1, 32	2.848	0.101	0.082	ns
SUVmean background	1, 32	7.127	0.012	0.182	s

The averaged background activity showed no significant correlation with the GFR, suggesting that the kidney uptake displays a specific accumulation rather than a non-specific one related to the poor kidney function and thereby longer circulation time due to deterioration of renal elimination.

**Fig. 3:**
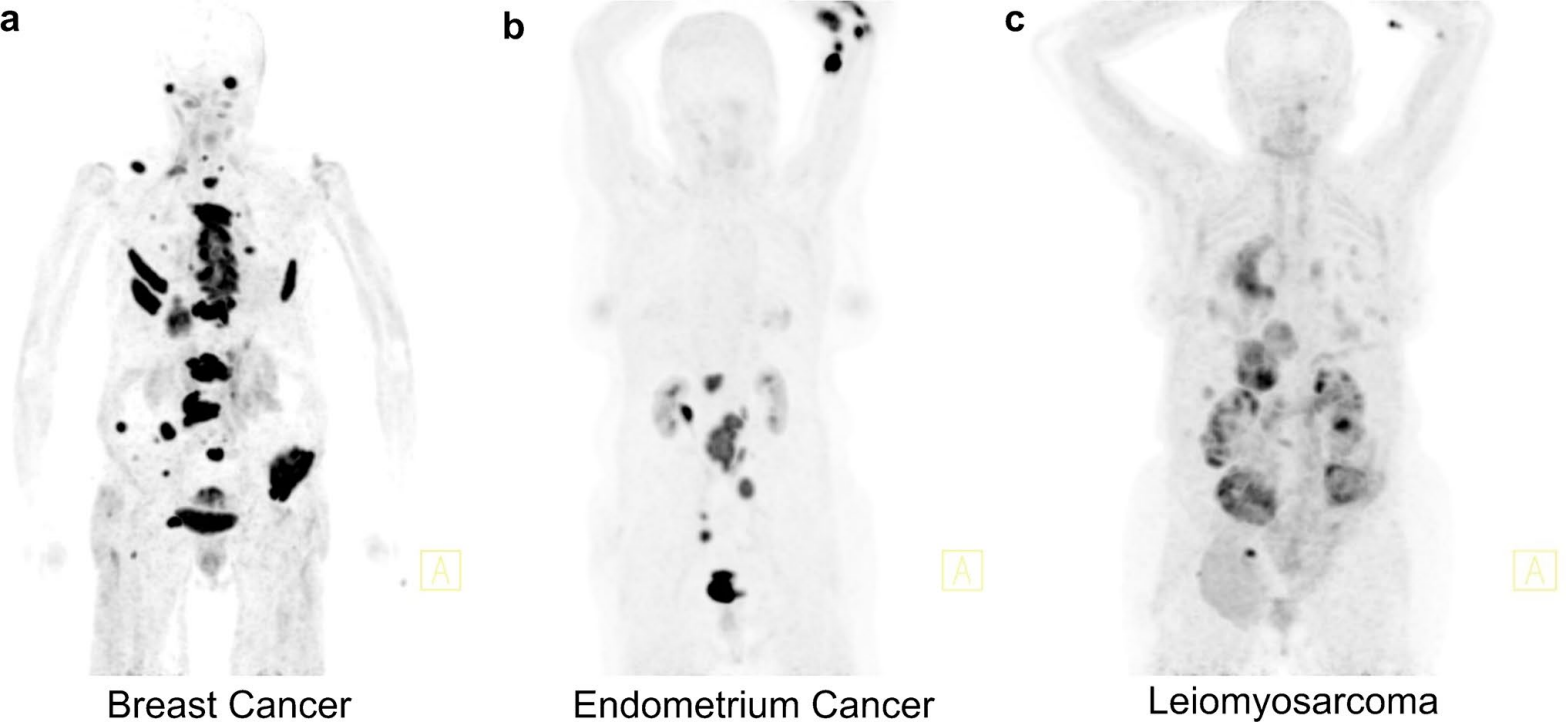
Comparison of 3 patients with different creatinine-levels undergoing a [^68^ Ga]Ga-FAPI PET/CT scan. Creatinine levels are **a** 78.68 µmol/l, **b** 108.73 µmol/l, and **c** 223.66 µmol/l. The tracer uptake of the kidney parenchyma visually correlates with the increased creatinine levels with a clear renal uptake in the leiomyosarcoma patient and no tracer uptake in the kidneys of the breast cancer patient. PET/CT scans were performed 1 h p.i. indicated by the specified tumor diagnosis (Figure 3a is adapted with permission from ref. 24)

Figure 3 illustrates the renal tracer uptake representatively in three different patients with either normal (a), moderately elevated (b), or severe elevated (c) creatinine levels.

### *[*^*68*^* Ga]Ga-DOTATOC-PET/CT*

Figure 2c and d depict the relationship between the averaged SUVmax and SUVmean values (upper pole, middle, lower pole) and the GFR of patients who underwent a [^68^ Ga]Ga-DOTATOC PET/CT scan. There was no significant correlation between tracer uptake of the kidneys and the GFR (R^2^ value of 0.134 and 0.049, respectively). For the SUVmax values and to a lesser extent also for the SUVmean values, the regression lines only exhibited a tendency that a lower GFR is associated with a slightly increased tracer uptake. Overall, however, there was a large spread of the measured values with the highest tracer uptake in a patient in CKD stage II. The averaged background values also showed no significant correlation with the GFR in the one-way ANOVA (Table 2).

### *[*^*68*^* Ga]Ga-PSMA-PET/CT*

Figure 2e and f illustrate the relationship between the averaged SUVmax or SUVmean values (upper pole, middle, lower pole) and the GFR of patients who received a [^68^ Ga]Ga-PSMA PET/CT scan. Both regression lines show no significant correlation between the tracer uptake of the kidneys and the GFR with generally very high SUVmax and SUVmean values of the kidneys (R^2^ value of 0.004 and 0.082, respectively). These results were confirmed by one-way ANOVA (Table 2). Surprisingly, the one-way ANOVA determined a significant correlation between the measured averaged background SUVmax and SUVmean values and the glomerular filtration values with higher SUVmax and SUVmean values associated with a poorer GFR.

### Comparison of the patient groups

In order to compare the group age, a Student’s *t* test was carried out between [^68^ Ga]Ga-FAPI and [^68^ Ga]Ga-PSMA as well as [^68^ Ga]Ga-FAPI and [^68^ Ga]Ga-DOTATOC. This comparison showed no significant difference regarding the age distribution within the groups ([^68^ Ga]Ga-FAPI/ [^68^ Ga]Ga-PSMA t-statistic: − 1.261, *p* (two tailed): 0.217; [^68^ Ga]Ga-FAPI/[^68^ Ga]Ga-DOTATOC t-statistic: 1.364, *p* (two tailed): 0.179).

## Discussion

[^68^ Ga]Ga-FAPI is an extremely interesting radiotracer with numerous possible clinical applications beyond oncological applications. The specific uptake of [^68^ Ga]Ga-FAPI in kidney fibrosis has been shown in a recent study in thirteen patients receiving renal puncture before the PET scan [8].

In the current study, the inverse correlation between GFR and [^68^ Ga]Ga-FAPI uptake is an indication for specific renal accumulation of the tracer in case of poor kidney function in patients with CKD, which is usually caused by progressive kidney fibrosis. Especially in comparison to the data collected for the two other evaluated radiotracers, we can conclude that the increased accumulation cannot simply be explained by a longer retention of the tracer in the parenchyma. Otherwise, we would expect an increased tracer accumulation also for [^68^ Ga]Ga-DOTATOC or [^68^ Ga]Ga-PSMA. In contrast to [^68^ Ga]Ga-DOTATOC [22] and [^68^ Ga]Ga-PSMA [23], only a very short residence time in the kidneys has been described for FAPI [24]. In comparison to Zhou et al., we could demonstrate in a larger cohort that a lower GFR correlates with a higher radiotracer uptake, independent of a known renal fibrosis. Another point that speaks against non-specific tracer accumulation by [^68^ Ga]Ga-FAPI is the lack of correlation between the background activities including the blood pool, measured in the abdominal aorta at the level of the renal arteries, the gluteus maximus muscle, the lung parenchyma, and the myocardium. This context suggests that fibroblasts involved in development of kidney fibrosis can be detected by FAP targeting. In contrast, [^68^ Ga]Ga-PSMA showed a significantly higher tracer accumulation in the organs used for the background activity with a poorer GFR. This confirms a certain unspecific binding or a prolonged tracer retention with poor kidney function. Due to very similar biodistribution patterns, [^68^ Ga]Ga-FAPI-04 and [^68^ Ga]Ga-FAPI-46 were analyzed in this study.

Despite the previously described specific binding of [^68^ Ga]Ga-PSMA in the kidney parenchyma, no correlation could be established between the GFR and the [^68^ Ga]Ga-PSMA uptake, although preclinical blocking studies have demonstrated the specific binding of PSMA [23]. A possible explanation for this would be that the PSMA-expressing proximal tubular cells of the kidneys do not decrease significantly in relation to the total number of cells in healthy kidneys when the kidney function is poor [25].

The comparability of the three patient groups is not significantly influenced by age and gender, as there is a similar age structure in a small patient collective, with the youngest average age for the [^68^ Ga]Ga-DOTATOC-PET/CTs (64 years) and the highest average age for the [^68^ Ga]Ga-PSMA-PET/CTs (73 years). A comparison of the age structure between [^68^ Ga]Ga-FAPI with [^68^ Ga]Ga-PSMA and [^68^ Ga]Ga-FAPI with [^68^ Ga]Ga-DOTATOC using the Student’s *t* test does not reveal any significant differences. In terms of gender distribution, there was a similar quotient between [^68^ Ga]Ga-DOTATOC (59% male) and [^68^ Ga]Ga-FAPI (75% male), while obviously only male patients were eligible for [^68^ Ga]Ga-PSMA-PET/CT. This creates a certain gender bias, but is not relevant for the interpretation of the obtained results. Visually, there was no significant difference in tracer uptake by the kidneys between the sexes in the different study groups. However, it has to be mentioned that the group of patients that underwent [^68^ Ga]Ga-FAPI-PET/CT scans is the smallest within the three groups and particularly the number of patients with a mildly decreased GFR value (60–89) is lower compared to the other groups (see Table 1). For future studies, higher availability of data on this specific subgroup would therefore be beneficial. Furthermore, it must be noted that the tracer amount available for kidney uptake may be reduced in case of disseminated oncologic disease. This so-called tumor sink effect [26] could affect the evaluation of CKD using [^68^ Ga]Ga-FAPI-PET/CT in patients with high tumor burden. Accordingly, future studies should also focus on the relevance of this phenomenon for patients suffering from CKD and oncologic disease.

## Conclusion

[^68^ Ga]Ga-FAPI has the potential to evaluate and quantify the grade of CKD noninvasively, whereas [^68^ Ga]Ga-DOTATOC or [^68^ Ga]Ga-PSMA uptake indicates no correlation to kidney function.

## Data Availability

The datasets analyzed during the current study are available from the corresponding author on reasonable request.
